# Effect of physical activity on asthma control in schoolchildren

**DOI:** 10.31744/einstein_journal/2020AO4936

**Published:** 2019-10-28

**Authors:** Ana Paula dos Santos, Márcio Júnior Strassburger, Cristian Roncada, Renato Tetelbom Stein, Paulo Márcio Pitrez, Simone Zeni Strassburger

**Affiliations:** 1 Universidade Regional do Noroeste do Estado do Rio Grande do Sul, Ijuí, RS, Brazil.; 2 Centro Universitário da Serra Gaúcha, Caxias do Sul, RS, Brazil.; 3 Pontifícia Universidade Católica do Rio Grande do Sul, Porto Alegre, RS, Brazil.

**Keywords:** Asthma, Exercise, Sedentary behavior, Child, Adolescent

## Abstract

**Objective:**

To investigate the effect of levels of physical activity on asthma control in children.

**Methods:**

A cross-sectional study, including public school students aged 8 to 12 years, of both sexes, with asthma, from a capital and a medium-sized cities in Southern Brazil. At home, the students answered the questionnaire on levels of physical activity and disease control.

**Results:**

A total of 482 schoolchildren with asthma participated in the study, with mean age of 10.9±2.2 years, and 253 (52%) were girls. Regarding disease control, 50% had controlled asthma, and 67% were considered sedentary. Schoolchildren with controlled asthma were more active than those with uncontrolled asthma (p=0.032). Active schoolchildren were more likely to have asthma controlled (OR=1.5; 95%CI: 1.04-2.25).

**Conclusion:**

The results demonstrated an association between physical activity levels and asthma control. More active schoolchildren were more likely to have asthma controlled.

## INTRODUCTION

Asthma is considered the most prevalent chronic disease in the pediatric age groups, affecting from 8.7 to 30.8% of this population in different Latin American countries.^( 1 )^ Due to its multifactorial etiology, compliance with treatment and control of the disease can be influenced by various factors.^( 1 , 2 )^ Absence of adequate control results in exacerbations and hospitalizations. In Brazil, there are approximately 110 hospitalizations for every 100 thousand inhabitants, and the mean hospital mortality rate is 0.46%.^( 3 , 4 )^

Currently, interest is growing in elucidating the influence of some factors, such as exposure to passive smoking, socioeconomic factors,^( 5 )^ obesity, and levels of physical activity in the control of asthma.^( 6 )^Many investigations around the world have addressed control of the disease, and physical activity seems to play an important role in the management of asthma.^( 7 )^

Reduction in the number of exacerbations and hospital admissions in asthmatic individuals with higher levels of physical activity has been reported,^( 8 )^besides others benefits, such as improved pulmonary function, control of inflammatory mediators, increase in aerobic capacity, and decrease in occurrence of bronchospasm induced by exercise in asthmatic individuals.^( 9 , 10 )^

Maintenance of asthma control helps in reducing the risk of exacerbations and hospitalizations, decreasing the use of health resources, and consequently, of costs of the disease (currently R$1.984,17/patient-year).^( 11 )^ However, scientific production regarding the relation between the levels of physical activity and asthma control in children and adolescents is still scarce, and the existing results are controversial.^( 12 , 13 )^

## OBJECTIVE

To investigate the effect of the levels of physical activity on the control of asthma.

## METHODS

This is an observational study about the prevalence and clinical characteristics of asthma in schoolchildren aged between 8 and 16 years, at a public education network. The study occurred in two distinct locations, and was conducted with schoolchildren of an industrialized metropolis (Porto Alegre, State of Rio Grande do Sul - RS), and schoolchildren from a medium-size city in the south of the country (Ijuí, a municipality located in the Northeast of the state of RS). Data were collected during a simultaneous period (2013-2014), applying the same cross-sectional design, in addition to the use of the same instruments for collection and analysis of the data. In Porto Alegre, 2,500 schoolchildren were evaluated, while in Ijuí, 1,688 schoolchildren were assessed. The study was submitted to the Research Ethics Committee of both locations, and was approved by Consolidated Opinion No. 73583/2012, CAAE: 03448512.0.0000.5336, and 131568/2012, CAAE: 08035412.5.0000.5336.

Data collection of both studies was organized based on two distinct phases. The first phase consisted of the selection of schoolchildren with asthma, based on the application of the instrument for epidemiological diagnosis of asthma, according to the International Study of Asthma and Allergies in Childhood (ISAAC) protocol.^( 14 )^ The instrument is composed of four questions that investigate medical diagnosis of asthma at any time of life, asthma crises over the previous 12 months, and at any time of life, in addition to the use of medications for asthma over the last 12 months. For the schoolchildren to be characterized as asthmatic, the four questions needed to have positive answers.

During the second phase, schoolchildren classified with asthma, as of triage of the first phase, answered control questionnaires about asthma and level of physical activity. Control of the disease was evaluated by means of the Asthma Control Test (ACT), with cut-off point at ≥20 points for categorization of controlled asthma.^( 15 )^ The levels of physical activity were investigated by a specific protocol, which calculated the weekly amount of time (in minutes) dedicated to physical activities. Patients were classified as sedentary schoolchildren if they had a sum of less than 300 minutes a week of reported physical activity.^( 16 )^

Analysis of the economic classification was based on the questionnaire *Critério de Classificação Econômica Brazil* (CCEB) [Brazil Economic Classification Criteria] of the *Associação Brasileira de Empresas de Pesquisa* (ABEP) [Brazilian Association of Research Companies].^( 17 )^

For statistical analysis, the continuous variables are presented as mean and standard deviation, or median and interquartile range, according to the symmetry of the variables. The categorical variables are presented as absolute and relative frequencies. For comparison of the variables with normal distribution among groups, Student’s *t* test was applied, and Mann-Whitney’s test was used for variables with abnormal distribution. Relations between the categorical variables were tested by the χ^2^ method, and the odds ratio between the variables was tested by the odds ratio method (OR). Differences between groups or analyses of outcomes were considered significantly different, with p<0.05. All the analyses were conducted using the Software (SPSS), version 18 (Chicago, USA).

## RESULTS

A total of 482 schoolchildren with asthma participated in the study (359 in Porto Alegre and 123 in Ijuí), with a mean age of 10.9±2.2 years, and 253 (52%) were girls. As to socioeconomic classification, 263 (75%) belonged to classes C, D, and E. As to control of asthma, 50.2% of participants presented with control of the disease. The prevalence of sedentary behavior was 67.8%; and 73.7% reported spending more than 2 hours daily in front of screens TV/cell phone/computer. The characterization data of the participating schoolchildren are shown on table 1 .

**Table 1:** Characterization of the schoolchildren with asthma

Characteristic	
Age	10.9±2.2
Female	253 (52.4)
Socioeconomic classification	
A and B	119 (24.7)
C, D and E	363 (75.3)
Controlled asthma	242 (50.2)
Sedentary behavior	327(67.8)
Screen time of more than 2 hours daily	355 (73.7)

Results expressed as mean±standard deviation or n (%).

Table 2 demonstrates the differences for the variables among schoolchildren, as per the level of physical activity. When we compare the schoolchildren who were asthmatic, active, and sedentary, we observed no difference between the groups for economic and screen time classification. Nevertheless, the frequency of active boys (64.5%) was greater when compared to that of girls (p<0.001), and schoolchildren with controlled asthma were more active (57.4%) than those with non-controlled asthma (p=0.032).

**Table 2:** Characterization of the variables according to the level of physical activity of the schoolchildren

Characteristic	Active n=155	Sedentary n=327	p value
Sex			
Female	55 (35.5)	188 (57.5)	<0.001*
Male	100 (64.5)	139 (42.5)	
Economic classification			
A and B	45 (29.0)	74 (22.6)	0.279
C, D and E	110 (71.0)	253 (77.4)	
Control of asthma			
Controlled	89 (57.4)	153 (46.8)	0.032*
Non-controlled	66 (42.6)	174 (53.3)	
Screen time			
More than 2 hours a day	110 (71.0)	245 (74.9)	0.377
Less than 2 hours a day	45 (29.0)	82 (25.1)	

Results expressed as n (%). *p<0.05 (χ^2^ test).

When we analyzed the effect of physical activities on control of asthma, as per table 3 , we found that active schoolchildren had a greater chance of presenting with controlled asthma (OR=1.5; 95%CI: 1.04-2.25). Screen time, sex, and the economic classification presented no significant relation with control of the disease.

**Table 3:** Relation of odds between asthma control and the variables assessed

	OR (95%CI)
Screen time	1.054 (0.703-1.581)
Sex	1.142 (0.799-1.633)
Level of physical activity	1.534 (1.043-2.255)*
Socioeconomic classification	1.265 (0.835-1.916)

* OR significant OR: odds ratio; 95%CI: 95% confidence interval.

## DISCUSSION

Recent studies have been conducted with the objective of knowing the impact that regular practice of physical activities can have on asthma. The present study evaluated the risk of this variable interfering in the control of the disease, resulting in physically more active schoolchildren presenting with a better control of asthma, thus reflecting a greater odds ratio of control of the disease.

There are few studies involving the pediatric age range aimed to evaluate the effect of the regular practice of physical activities on asthma control. Matsunaga et al.,^( 12 )^ investigated asthmatic children and adolescents, and correlated the level of control of asthma and levels of physical activity during the previous week, and found no significant associations between them. In another study, it was found that those who were physically active were also more inclined to report symptoms of asthma during the day and night awakenings.^( 13 )^

A study done with 643 adult asthmatics reached results similar to ours, considering that the total time of habitual physical activities was related to the control of the asthma, and the probability of obtaining good control of the disease was twice as high in active asthmatics. Its findings highlighted that in the highest quartile of physical activity were almost 2.5 times more controlled (OR=2.47) in comparison with those who did not get involved in any type of physical activity.^( 18 )^

In another study involving 61 adults with severe asthma, it was noted that higher indices of physical activities and lower levels of sedentary behavior are associated with a better capacity for exercise, better control of inflammatory mediators, and better control of the disease.^( 19 )^

Despite recent studies pointing towards the regular practice of physical activities as an important factor for asthma control, some results still seem controversial. Verlaet et al.,^( 20 )^ found the regular practice of moderate intensity physical activities was considered a factor for the control of asthma in men, while the regular practice of rigorous intensity physical activities, increased the risk of uncontrolled asthma in women. The study by Lövström et al.,^( 21 )^ reported women with high levels of regular physical activity, as compared to moderate levels of activity, would have less control of asthma. Thus, it seems that the practice of regular physical activities with moderate intensity would be more protective against the symptoms of asthma than activities with vigorous intensity. Del Giacco et al.,^( 22 )^ reinforced the fact that moderate levels of regular physical activities have a positive effect on the control of asthma.

Therefore, physical inactivity may be associated with a low index of control of the disease. Additionally, asthmatic children tend to be more physically inactive when compared to healthy children.^( 23 , 24 )^

We identified a high rate of sedentary behavior (67.8%) among the schoolchildren of the present study. Roncada et al.^( 25 )^ found a similar result: 67.2% of asthmatic children were sedentary. Teng et al.,^( 26 )^ one the other hand, noted greater physical inactivity (less than 300 minutes a week) in girls when compared to boys, corroborating the results of the present study, according to which the frequency of active boys was greater when compared to girls (p<0.001).

Remaining for a prolonged time in front of screens is considered detrimental to health.^( 27 )^In the present study, 73% of asthmatic individuals remained more than 2 hours a day in screen time. Willeboordse et al.,^( 28 )^ found lower levels of physical activity in school-aged children who spent more sedentary minutes in screen time than being physically active. Physical inactivity is one of the primary risk factors for morbidity and mortality, has great influence on the prevalence of non-communicable diseases, and it is considered a serious public health problem.^( 29 )^

Asthma control is essential for a good quality of life, and is considered the primary objective of treatment.^( 1 )^ Of the study schoolchildren, only 50% presented with controlled asthma. Gazzotti et al.,^( 30 )^ also noted low levels of controlled asthma (9.3%) and high levels of partially controlled (56.5%) and non-controlled (32.2%) asthma. In addition to the lack of control of asthma, the disease is associated with an increased number of hospitalizations.

Some limitations should be considered in the present study. The scarcity of other studies that related the outcome variables in the pediatric population hindered a more consistent discussion. The population of schoolchildren originated from two studies with the same methodology, but carried out in different cities, with distinct regional characteristics. This is understood as not disqualifying the analysis of the outcome variables investigated. The test applied, such as ACT and the level of physical activity, are validated, although self-reported, containing possible biases of answers inherent to this type of tool. The instrument utilized did not include questions relative to the intensity of the physical activities, preventing the analysis of outcomes for this variable. Finally, since this is a cross-sectional design, it is impossible to define the exact direction of the relation between control of asthma and physical activity. Our results probably reflect the possibility that those with better asthma control were engaged in more physical activities.

## CONCLUSION

The results demonstrate an association between the levels of satisfactory physical activity and acceptable levels of asthma control. The most active schoolchildren presented with higher odds ratios of having controlled asthma. Additionally, the sedentary lifestyle proved to be present in a large part of the study schoolchildren. We understand that it is necessary to expand the studies about the possible effects of regular practice of physical activities for control of asthma in this age range. Moreover, it is important to further understand this regular practice as a form of supporting treatment for children and adults with asthma, seeking to improve the control rates of the disease.
