# Supplementary material for: Effect of physical activity on asthma control in schoolchildren
Source: Einstein (Sao Paulo). 2019 Oct 28;18:eAO4936. doi: 10.31744/einstein_journal/2020AO4936 (PMC6896598; doi:10.31744/einstein_journal/2020AO4936)
Supplement: Translation

# Translation: Efeito da atividade física no controle da asma em escolares

**Authors:** Ana Paula dos Santos, Márcio Júnior Strassburger, Cristian Roncada, Renato Tetelbom Stein, Paulo Márcio Pitrez, Simone Zeni Strassburger

**Affiliations:** 1 Universidade Regional do Noroeste do Estado do Rio Grande do Sul, Ijuí, RS, Brasil.; 2 Centro Universitário da Serra Gaúcha, Caxias do Sul, RS, Brasil.; 3 Pontifícia Universidade Católica do Rio Grande do Sul, Porto Alegre, RS, Brasil.

**Keywords:** Asma, Exercício, Comportamento sedentário, Criança, Adolescente

## Abstract

**Objetivo:**

Investigar o efeito dos níveis de atividade física no controle da asma em crianças.

**Métodos:**

Estudo transversal, incluindo escolares da rede pública, de 8 a 12 anos, de ambos os sexos, com asma, de uma capital e de uma cidade de porte médio da Região Sul do Brasil. Os escolares responderam, em seus domicílios, um questionário de níveis de atividade física e de controle da doença.

**Resultados:**

Participaram da pesquisa 482 escolares com asma, com média de idade de 10,9±2,2 anos, e 253 (52%) eram meninas. Quanto ao controle da doença, 50% apresentavam asma controlada e 67% foram considerados sedentários. Os escolares com asma controlada foram mais ativos do que os com asma não controlada (p=0,032). Os escolares ativos tiveram mais chance de ter a asma controlada (RC=1,5; IC95%: 1,04-2,25).

**Conclusão:**

Os resultados demonstraram associação entre os níveis de atividade física e controle da asma. Os escolares mais ativos apresentaram mais chance de ter a asma controlada.

## INTRODUÇÃO

A asma é considerada a doença crônica mais prevalente da faixa etária pediátrica, atingindo de 8,7 a 30,8% dessa população em diferentes países da América Latina.^( 1 )^Devido à sua etiologia multifatorial, a aderência ao tratamento e o controle da doença podem ser influenciados por diversos fatores.^( 1 , 2 )^A ausência de controle adequado resulta em exacerbações e hospitalizações. No Brasil, ocorrem aproximadamente 110 hospitalizações para cada 100 mil habitantes, e taxa média de mortalidade hospitalar é de 0,46%.^( 3 , 4 )^

Atualmente, é crescente o interesse em elucidar a influência de fatores, como exposição ao tabagismo passivo, fatores socioeconômicos,^( 5 )^obesidade e níveis de atividade física no controle da asma.^( 6 )^O controle da doença tem sido alvo de pesquisas em todo o mundo, e a atividade física parece ter papel importante no manejo da asma.^( 7 )^

A redução do número de exacerbações e das internações em indivíduos asmáticos com maiores níveis de atividade física é relatada,^( 8 )^além de outros benefícios, como melhora da função pulmonar, controle de mediadores inflamatórios, aumento da capacidade aeróbica, e diminuição da ocorrência de broncoespasmo induzido por exercício, em indivíduos asmáticos.^( 9 , 10 )^

A manutenção do controle da asma auxilia na redução do risco de exacerbações e hospitalizações, diminuindo a utilização de recursos de saúde e, consequentemente, dos custos da doença (atualmente R$1.984,17/paciente-ano).^( 11 )^Contudo, a produção científica a respeito da relação entre os níveis de atividade física e controle da asma em crianças e adolescentes ainda é escassa, e os resultados existentes são controversos.^( 12 , 13 )^

## OBJETIVO

Investigar o efeito dos níveis de atividade física no controle da asma.

## MÉTODOS

Trata-se de estudo observacional sobre prevalência e características clínicas da asma em escolares da rede pública de ensino, com idade entre 8 e 16 anos. A pesquisa ocorreu em dois locais distintos, tendo sido realizada com escolares de uma metrópole industrializada (Porto Alegre, RS) e escolares de uma cidade de porte médio do sul do país (Ijuí, município localizado no noroeste do Estado do RS). As coletas de dados ocorreram em período simultâneo (2013-2014), aplicando o mesmo desenho metodológico transversal, além do emprego dos mesmos instrumentos de coleta e análise dos dados. Em Porto Alegre, avaliaram-se 2.500 escolares, enquanto em Ijuí, foram avaliados 1.688 escolares. A pesquisa foi submetida aos Comitês de Ética em Pesquisa de ambos os locais, sendo aprovada sob pareceres consubstanciados 73583/2012, CAAE: 03448512.0.0000.5336 e 131568/2012, CAAE: 08035412.5.0000.5336.

A coleta dos dados de ambas as pesquisas foi organizada a partir de duas etapas distintas. A primeira etapa consistiu na triagem dos escolares com asma, a partir da aplicação do instrumento para o diagnóstico epidemiológico de asma, de acordo com o protocolo *International Study of Asthma and Allergies in Childhood* (ISAAC).^( 14 )^O instrumento é composto por quatro questões, que investigam diagnóstico médico de asma alguma vez na vida, crises de asma nos últimos 12 meses e em algum momento na vida, além da utilização de medicamentos para asma nos últimos 12 meses. Para que os escolares fossem caracterizados como asmáticos, as quatro perguntas deveriam ter respostas positivas.

Na segunda etapa, os escolares classificados com asma, a partir da triagem da primeira etapa, responderam questionários de controle de asma e nível de atividade física. O controle da doença foi avaliado por meio do Teste de Controle de Asma (ACT - *Asthma Control Test* ) com ponto de corte em ≥20 pontos para categorização da asma controlada.^( 15 )^Os níveis de atividade física foram investigados por protocolo específico, que contabilizou a quantidade de tempo semanal (em minutos) dedicada a atividades físicas. Foram classificados como escolares sedentários aqueles com menos de 300 minutos semanais acumulados de atividade física relatada.^( 16 )^

A análise da classificação econômica foi realizada com base no questionário Critério de Classificação Econômica Brasil (CCEB), da Associação Brasileira de Empresas de Pesquisa (ABEP).^( 17 )^

Para fins de análise estatística, as variáveis contínuas são apresentadas por média e desvio padrão, ou mediana e intervalo interquartil, conforme a simetria das variáveis. As variáveis categóricas estão apresentadas por frequências absolutas e relativas. Para comparação das variáveis, com distribuição normal entre grupos, foi aplicado o teste *t* de Student, e o teste de Mann-Whitney foi usado para as variáveis com distribuição anormal. As relações entre as variáveis categóricas foram testadas pelo método de χ^2^, e a razão de chances entre as variáveis foi testada pelo método de razão de chances (RC). As diferenças entre grupos ou análises de desfecho foram consideradas significativamente diferentes com p<0,05. Todas as análises foram realizadas usando Software (SPSS), versão 18 (Chicago, EUA).

## RESULTADOS

Participaram do estudo 482 escolares com asma (359 em Porto Alegre e 123 em Ijuí), com média de 10,9±2,2 anos, e 253 (52%) eram meninas. Quanto à classificação socioeconômica, 263 (75%) pertenciam às classes C, D e E. Quanto ao controle da asma, 50,2% dos participantes apresentaram controle da doença. A prevalência de sedentarismo foi de 67,8% e 73,7% relataram permanecer mais de 2 horas diárias frente às telas TV/celular/computador. Os dados de caracterização dos escolares participantes encontram-se na tabela 1 .

**Tabela 1:** Caracterização dos escolares com asma

Característica	
Idade	10,9±2,2
Sexo feminino	253 (52,4)
Classificação socioeconômica	
A e B	119 (24,7)
C, D e E	363 (75,3)
Asma controlada	242 (50,2)
Sedentários	327(67,8)
Mais de 2 horas diárias na frente de telas	355 (73,7)

Resultados expressos por média±desvio padrão ou n (%).

A tabela 2 demonstra as diferenças para as variáveis entre escolares, de acordo com o nível de atividade física. Quando comparamos os escolares com asma, ativos e sedentários, não observamos diferença entre os grupos para a classificação econômica e tempo em frente a telas. Porém, a frequência de meninos ativos (64,5%) foi maior quando comparada à das meninas (p<0,001), e os escolares com asma controlada foram mais ativos (57,4%) do que os com asma não controlada (p=0,032).

**Tabela 2:** Caracterização das variáveis de acordo com o nível de atividade física dos escolares

Característica	Ativo n=155	Sedentário n=327	Valor de p
Sexo			
Feminino	55 (35,5)	188 (57,5)	<0,001*
Masculino	100 (64,5)	139 (42,5)	
Classificação econômica			
A e B	45 (29,0)	74 (22,6)	0,279
C, D e E	110 (71,0)	253 (77,4)	
Controle da asma			
Controlada	89 (57,4)	153 (46,8)	0,032*
Não controlada	66 (42,6)	174 (53,3)	
Tempo na frente de telas			
Mais de 2 horas diárias	110 (71,0)	245 (74,9)	0,377
Menos de 2 horas diárias	45 (29,0)	82 (25,1)	

Resultados expressos por n (%). *p<0,05 (teste do χ^2^).

Quando analisamos o efeito das atividades físicas sobre o controle da asma, de acordo com a tabela 3 , encontramos que os escolares ativos tiveram maior chance de apresentar a asma controlada (RC=1,5; IC95%: 1,04-2,25). O tempo em frente a telas, sexo e a classificação econômica não apresentaram relação significativa com o controle da doença.

**Tabela 3:** Relação de chance entre o controle da asma e as variáveis avaliadas

	RC (IC95%)
Tempo na frente de telas	1,054 (0,703-1,581)
Sexo	1,142 (0,799-1,633)
Nível de atividade física	1,534 (1,043-2,255)*
Classificação socioeconômica	1,265 (0,835-1,916)

* RC significativa. RC: razão de chance; IC95%: intervalo de confiança de 95%.

## DISCUSSÃO

Pesquisas recentes estão sendo realizadas com o intuito de conhecer o impacto que a prática regular de atividades físicas pode causar na asma. O presente estudo avaliou o risco dessa variável interferir no controle da doença, tendo como resultado que escolares fisicamente mais ativos apresentam melhor controle da asma, refletindo em uma maior razão de chances de controle da doença.

Existem poucos estudos envolvendo a faixa etária pediátrica que têm como objetivo avaliar o efeito da prática regular de atividades físicas no controle da asma. Matsunaga et al.,^( 12 )^pesquisaram crianças e adolescentes asmáticos e correlacionaram o nível de controle da asma e os níveis de atividade física praticados na última semana, não encontrando associações significativas entre elas. Em outro estudo, encontrou-se que aquelas que eram fisicamente ativas, também eram mais propensas a relatar sintomas de asma durante o dia e despertares noturnos.^( 13 )^

Um estudo com 643 asmáticos adultos chegou a resultados semelhantes aos nosso, visto que o tempo total de atividades físicas habitual foi relacionado com o controle da asma, e a probabilidade de obter um bom controle da doença era duas vezes maior em asmáticos ativos. Seus achados destacam que aqueles no quartil mais alto de atividade física eram quase 2,5 vezes mais controlados (OR=2,47) em comparação com aqueles que não se envolveram em qualquer atividade física.^( 18 )^

Em outro estudo envolvendo 61 adultos com asma grave, observou-se que níveis mais altos de atividades físicas e níveis mais baixos de sedentarismo estão associados a melhor capacidade ao exercício, melhor controle de mediadores inflamatórios e melhor controle da doença.^( 19 )^

Apesar de estudos recentes apontarem a prática regular de atividades físicas como fator importante para controle de asma, alguns resultados ainda parecem ser controversos. Verlaet et al.,^( 20 )^encontraram que a prática regular de atividades físicas de intensidade moderada foi considerada fator de controle de asma em homens, enquanto a prática regular de atividades físicas, com intensidade rigorosa aumentou o risco de asma não controlada em mulheres. O estudo de Lövström et al.,^( 21 )^relatou que mulheres com alto níveis de atividade física regulares, em comparação com níveis moderados de atividades, teriam menor controle da asma. Assim, parece que a prática de atividades físicas com intensidade moderada regular seria mais protetora contra os sintomas da asma do que as atividades com intensidade vigorosa. Del Giacco et al.,^( 22 )^reforçaram que níveis moderados de atividades físicas regulares possuem um efeito positivo no controle da asma.

Dessa forma, a inatividade física pode estar associada ao baixo índice de controle da doença. Além disso, crianças asmáticas tendem a ser mais fisicamente inativas quando comparada às crianças saudáveis.^( 23 , 24 )^

Encontramos alta taxa de sedentarismo (67,8%) entre os escolares do presente estudo. Roncada et al.,^( 25 )^encontraram resultado semelhante: 67,2% das crianças asmáticas eram sedentárias. Teng et al.,^( 26 )^por sua vez, observaram maior inatividade física (menos de 300 minutos por semana) em meninas em comparação aos meninos, corroborando os resultados do presente, segundo os quais a frequência de meninos ativos era maior quando comparado as meninas (p<0,001).

Permanecer por tempo prolongado em frente às telas é considerado prejudicial à saúde.^( 27 )^No presente estudo, 73% dos asmáticos permaneciam mais de 2 horas diárias em frente às telas. Willeboordse et al.,^( 28 )^encontraram baixos níveis de atividade física em crianças em idade escolar, que passavam mais minutos sedentários atrás das telas do que sendo fisicamente ativas. A inatividade física é um dos principais fatores de risco para a morbimortalidade, e tem grande influência na prevalência de doenças não transmissíveis, sendo considerado grave problema de saúde pública.^( 29 )^

O controle da asma é essencial para uma boa qualidade de vida, considerado objetivo primário do tratamento.^( 1 )^Dos escolares do estudo, somente 50% apresentaram ter asma controlada. Gazzotti et al.,^( 30 )^também notaram níveis baixos de asma controlada (9,3%) e níveis altos de asma parcialmente controlada (56,5%) e não controlada (32,2%), além de que a falta de controle da asma está associada ao aumento do número de hospitalizações.

Algumas limitações devem ser consideradas no presente estudo. A escassez de outros estudos que relacionem as variáveis de desfecho na população pediátrica dificultou uma discussão mais consiste. A população de escolares originou-se de dois estudos com a mesma metodologia, contudo, realizados em cidades diferentes, com características regionais distintas. Entende-se que isso não desqualifica a análise das variáveis de desfecho pesquisadas. Os testes aplicados, como ACT e nível de atividade física, são validados, porém, autorrelatados, contendo possíveis vieses de respostas inerentes a este tipo de instrumento. O instrumento utilizado não incluiu perguntas relativas à intensidade das atividades físicas, impossibilitando a análise dos desfechos para esta variável. Por fim, como trata-se de delineamento transversal, é impossível definir a direção exata da relação entre o controle da asma e a atividade física. É provável que nossos resultados reflitam a possibilidade de que aqueles com melhor controle da asma se envolvam em mais atividade física.

## CONCLUSÃO

Os resultados demonstram associação entre níveis de atividade física satisfatórios e níveis aceitáveis de controle da asma. Os escolares mais ativos apresentaram maior razão de chances de ter a asma controlada. Além disso, o estilo de vida sedentário mostrou-se presente em grande parte dos escolares do estudo. Entendemos ser necessário ampliar os estudos sobre os possíveis efeitos da prática regular de atividades físicas para o controle da asma nessa faixa etária, bem como aumentar o entendimento desta prática regular como forma tratamento coadjuvante para crianças e adultos com asma, visando melhorar as taxas de controle da doença.
